# The problematic of soil contamination by industries in a protected area in Portugal

**DOI:** 10.1007/s10653-025-02445-5

**Published:** 2025-04-09

**Authors:** Fernando Michels, Joana Ribeiro, Maria Helena Henriques

**Affiliations:** 1 Universidade de Coimbra, Departamento de Ciências da Terra da Universidade de Coimbra, Rua Sílvio Lima, 3030-790 Coimbra, Portugal; 2 Universidade de Coimbra, Centro de Geociências da Universidade de Coimbra, Rua Sílvio Lima, 3030-790 Coimbra, Portugal; 3 Universidade de Coimbra, Instituto Dom Luiz - Polo da Universidade de Coimbra, Rua Sílvio Lima, 3030-790 Coimbra, Portugal

**Keywords:** Cabo Mondego (Portugal), Potentially toxic elements, Soil quality, Cement industry, Coal combustion, Contamination sources

## Abstract

Understanding the environmental legacy surrounding abandoned industrial sites, once powered by fossil fuels and operated in times of less stringent environmental regulations, is crucial today. This issue is especially pertinent in regions that have since become protected areas. Cabo Mondego, recognized internationally for its geological diversity, designated as a National Monument of Portugal, and included in the Atlantic Geopark project, represents such a region due to its history of potentially degrading activities associated with coal mining and an industrial complex focused on cement and lime production. To ensure the sustainable use of this protected area, it is essential to assess soil health. In this study, fifty soil samples were collected from areas bordering the decommissioned industrial complex and coal mining remnants. The research detected contaminants, their geochemical associations, and potential sources. The evaluation included comparisons with reference values, assessment of the contamination severity, multivariate data analysis, and spatial distribution analysis. Three primary anthropogenic sources of soil contamination were identified: (1) atmospheric emissions from the industrial complex, primarily linked to As and Se contamination, with additional samples showing contamination by Ni, V, and Cr; (2) localized coal mining waste disposal, associated with As, Mo, and Se contamination; and (3) isolated fuel contamination, indicated by elevated Pb levels. Additionally, the study suggests that geogenic sources contribute to the elevated As and U levels in soils with specific geological characteristics. This characterization of potential toxic contaminants in Cabo Mondego enhances the understanding of the region's vulnerabilities and highlights the importance of its protection.

**Supplementary Information:**

The online version contains supplementary material available at 10.1007/s10653-025-02445-5.

## Introduction

Protected areas are crucial for safeguarding valuable natural resources and ensuring their preservation for future generations. Globally, instances where protected areas have a history of industrial and mining activities are rare, but these sites present unique challenges for environmental management. In such locations, soil investigations are particularly important for assessing the environmental legacy and guiding strategies to ensure the long-term protection of these ecosystems.

The enrichment of potentially toxic elements (PTE) in soils from protected areas has been assessed globally, as evidenced by several studies (Kurt et al., 2024; Mazurek et al., 2017; Staszewski et al., 2012; Tomaskin et al., 2013). In each case, the distribution of PTE and the degree of contamination is highly dependent on the local soil's physicochemical properties, parent materials and the characteristics of the terrain. In general, the highest content of PTE occurs in surface soils (Kabata-Pendias, 2011). Since the last century, anthropogenic activities have been identified as the primary sources of PTE in soils from national parks (Mazurek et al., 2017; Ratko et al., 2011). These sources can be distinguished as ex-situ and in-situ influences. Among the ex-situ activities, notable contributors include mining operations, the disposal of urban waste, high-traffic roads, and intensive agricultural practices. In-situ influences encompass excessive tourism and the overexploitation of natural resources.

Cabo Mondego, located on the central Portuguese Atlantic coast, exemplifies such a region of a protected area with a industrial past. Distinguished by its geological heritage of international relevance and its remarkable landscape, Cabo Mondego hosts a Global Boundary Stratotype Section and Point (GSSP) for the Bajocian Stage (Middle Jurassic), an Auxiliary Section and Point (ASSP) for the Bathonian Stage (Middle Jurassic), and other geosites of significant scientific value (Henriques, 1998; Henriques et al., 1994; Rocha et al., 2014). Currently, Cabo Mondego is part of Portugal's National Network of Protected Areas and is designated as a Natural Monument (Decreto Regulamentar n.º 82/2007 de 3 de outubro, 2007). Furthermore, it is involved in the ongoing Atlantic Geopark Project (Custódio et al., 2024; Vaz et al., 2023), with the goal of joining the United Nations Educational, Scientific, and Cultural Organization (UNESCO) Global Geoparks Network.

Adjacent to the classified area of the Cabo Mondego Natural Monument, there is a deactivated industrial complex that ceased operations in 2013, which hosted several types of industries for over a century with emphasis on the production of hydraulic lime and cement (Goulão, 2016), along with mining of limestone in three quarries that supplied raw materials to the industrial complex. The site also hosted one of Portugal’s first coal mines (Pinto et al., 2014), which was ended operation in the latter half of the twentieth century. According to Campos (1983), the lime industry used coal extracted in Cabo Mondego mine, together with coal from other portuguese coal mines as its primary fuel for heating the kilns for a significant period of operation, at least until the end of the 1980s. This industry was decommissioned in 2013. In contrast, the cement factory was fueled with fuel oil from the 1960s until its closure in 1986 (Campos, 1983). Nowadays, remnants of the mining operations can be observed on the territory such as gallery entrances, coal mining wastes, part of the infrastructure of the industrial facilities, as well as others deactivated administrative buildings.

The industrial activities mentioned are known as sources of PTE contaminating land and water ecosystems, and impacting living organisms (Cutillas-Barreiro et al., 2016; Rouhani et al., 2023). PTE refer to a group of elements that includes metals (e.g., Cd, Mo, Hg, Pb), metalloids (e.g., As), and non-metals (e.g., Se) that are found naturally in the environment but may be toxic for flora and fauna when their concentrations exceed certain thresholds (Thalassinos et al., 2023). The ingress of PTE into the soil environment can occur through natural processes such as rock weathering and pedogenesis, or via anthropogenic sources (Antoniadis et al., 2019; Silva et al., 2021; Weissmannová & Pavlovský, 2017).

The presence of PTE in soils originating from anthropogenic sources emerges as a significant environmental concern (Ameraoui et al., 2017; Ismail et al., 2023; Rouhani et al., 2023). This concern is related to PTE toxicity and persistence in the environment coupled with their propensity for bioaccumulation depending on the soil physical–chemical properties (Cutillas-Barreiro et al., 2016; Tack, 2010). Several studies have addressed research of PTE contamination in soils and sediments surrounding cement and lime plants (e.g., Cutillas-Barreiro et al., 2016; Jafari et al., 2019; Parlak et al., 2023; Silva et al., 2021; Tack, 2010). The characteristics of the emission levels of contaminants depend on the industries technological and operational conditions and can occur in several stages of production, from the raw material extraction and management to the combustion process necessary to heat the furnaces (Cutillas-Barreiro et al., 2016). Achternbosch et al. (2003) enumerate elements introduced into cement via raw materials, including As, Cd, Co, Cr, Cu, Mn, Ni, Pb, Se, U, V, and Zn. After the emission into the atmosphere, part of the contaminants is deposited in soils and water courses by dry or wet deposition. The extent of the affected areas depends on factors such as the type and density of contaminating elements and meteorological parameters (principally wind and precipitation) (Rothenberg et al., 2010; Semhi et al., 2010). The contaminants end up accumulating on surfaces around industrial areas and can impact the environment and human health.

In the absence of specific data regarding soil contamination near cement and lime industries in Portugal, a systematic compilation of data obtained from international studies was performed (Supplementary Material A). These studies have documented that Cd, Co, Cr, Cu, Mn, Ni, Pb, and Zn are associated with environmental problems in the vicinity of cement factories (Ameraoui et al., 2017; Ismail et al., 2023; Jafari et al., 2019; Kaur et al., 2019; Khwedim et al., 2015; Kolo et al., 2018; Parlak et al., 2023; Silva et al., 2021), highlighting a significant environmental issue associated with this type of industry.

Regarding soil contamination around coal mines, many PTE released into the environment may be generated during various stages of the coal mining process, such as transportation, leachates from waste disposal and wastewater treatment (Qiao et al., 2011; Rouhani et al., 2023). Identifying the sources of PTE in soils of coal mining areas is often challenging, as they may originate not only from emissions during coal extraction and wastes disposal but also from atmospheric deposition from coal-fired power plants and dispersion during other coal industry-related operations, which frequently are located nearby. Studies examining PTE concentration near coal mining sites globally indicate that the most frequently analyzed elements include As, Cd, Cr, Cu, Hg, Ni, Pb, and Zn (Boahen et al., 2022; Boente et al., 2018; Rouhani et al., 2023; Yan et al., 2022; Zhang et al., 2021).

The absence of data about soil quality and concentration of PTE in the Cabo Mondego territory underscores the importance of this investigation. The objectives of this study are to assess the soil quality and the extent of environmental contamination in the area, identifying types, sources, and distribution of contaminants. This research aims to contribute to a comprehensive understanding of soil quality and health in Cabo Mondego, ultimately enabling competent authorities to make informed technical and policy decisions for the protection of the region. The significance of this study lies in its potential to shed light on the contamination of soils, offering crucial insights for environmental management to safeguard the ecological integrity of the area.

## Materials and methods

### Study area and sampling

**Fig. 1:**
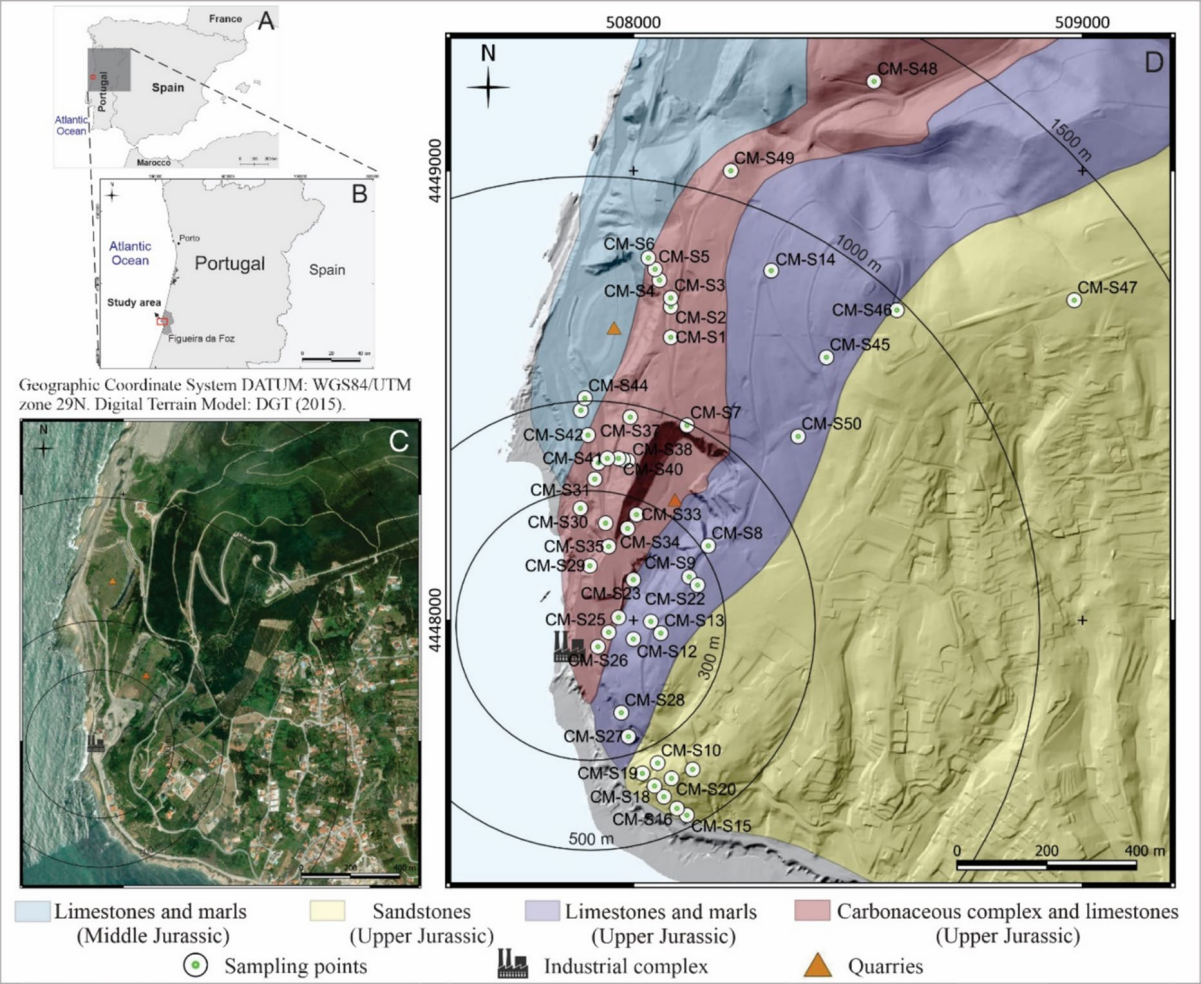
Location map of the study area within Iberian Peninsula and Portugal (**A** and **B**, respectively), a local satellite image (**C**), and geological map (modified from Rocha et al., 1976) with identification of sampling points (**D**). Digital Terrain Model (DGT, 2015)

The research was carried out in an emerged area of approximately 0.42 km^2^ that belongs to the municipality of Figueira da Foz (Fig. 1). The geology of the studied area is dominated by Jurassic formations of limestones, marls, sandstones and carbonaceous materials, including coal seams, from the northern sector of Lusitanian Basin. The Middle Jurassic sedimentary record is exposed in the northern sector of the study area, represented by the Cabo Mondego Formation, comprising limestone, marly limestone, and marls (Fig. 1). In the remaining study area, the Upper Jurassic succession is characterized by limestones, marls and coal layers from the Carbonaceous complex of the Vale Verde Formation (targeted for coal exploitation), along with sandstones from the Boa Viagem Formation (Pena dos Reis, 2006; Rocha et al., 1981; Trincão et al., 2018).

Regarding land use management instruments, the municipality of Figueira da Foz (PDM, 2017) classifies the territory as: "Cultural Spaces", encompassing the area of the industrial complex where the rehabilitation of buildings and surrounding exterior spaces is permitted for tourism, leisure, research and development purposes; "Natural Spaces" to the north of the industrial facilities where the areas are sensitive from ecological, landscape, and environmental perspectives, prioritizing the protection, conservation, rational management, renewal capacity of natural resources, and safeguarding of landscape values; "Protective Forest Spaces" to the east; and "Mixed Use Forest-Agricultural Spaces" to the south of the industrial complex.

According to the Köppen climate classification, Cabo Mondego is situated in a temperate climate zone with dry, mild summers (IPMA, 2024; Kottek et al., 2006). Precipitation patterns are irregular throughout the year, with the summer months being the driest, while December and January have the highest average precipitation levels. The occurrence of winds is constant and evenly distributed throughout the year. The northern quadrant shows the highest frequency of flow, followed by the southern and eastern quadrants (Rocha, 2010; SNIRH, 2024).

Soils in the area are classified as calcic cambisols according to the Soil Map of Portugal (Cardoso, 1974). These soils are characterized by slightly developed horizon differentiation in the subsoil and are strongly drained with incipient stage of soil formation, in this case categorized as a mineral-origin soils with an ochric A horizon and a calcic horizon (FAO, 2015; SNIAMB, 2024).

**Fig. 2:**
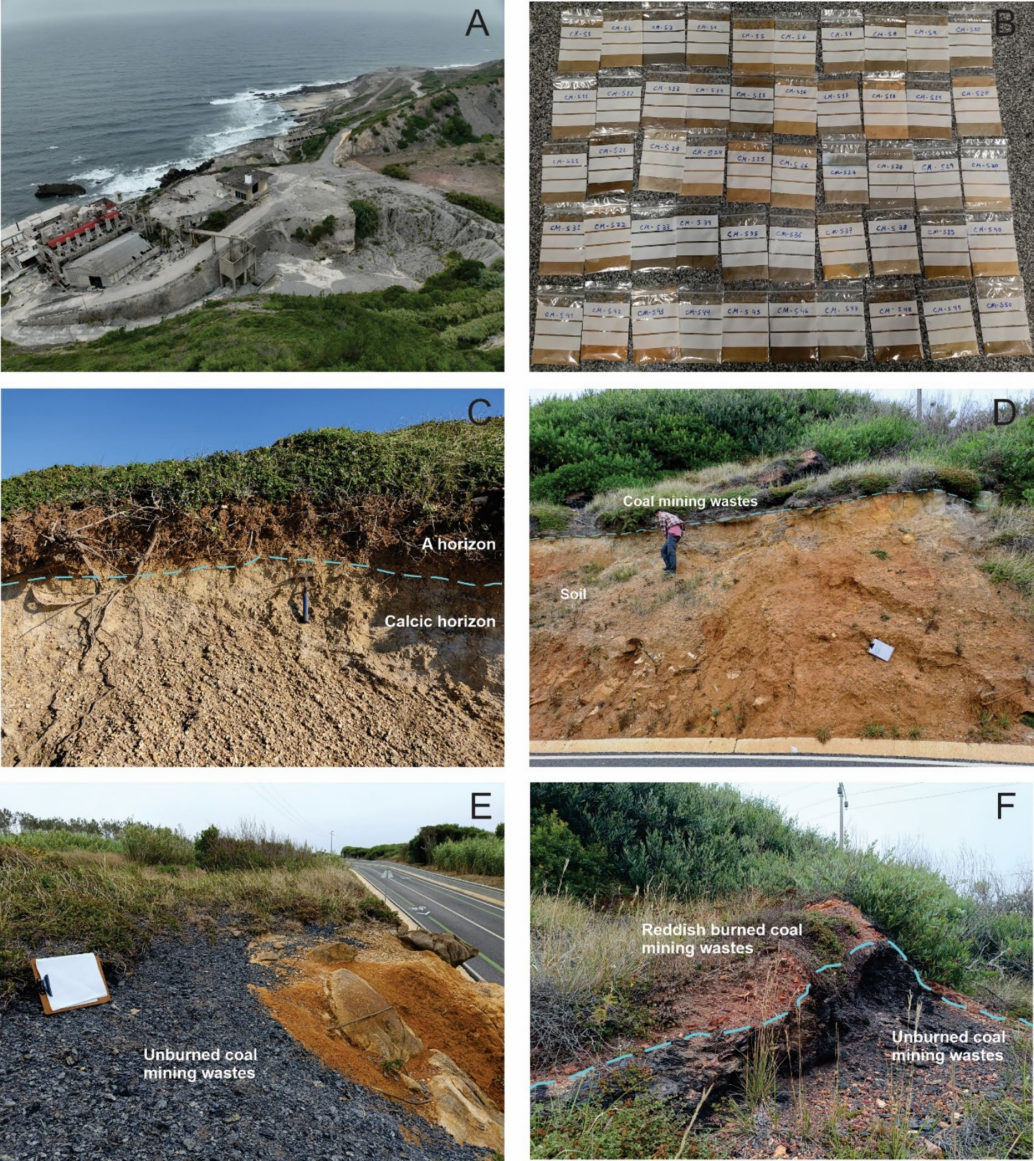
**A**. Built-up areas of the deactivated industrial complex at Cabo Mondego; **B**. Soil samples after laboratorial processing where is possible to observe heterogeneous colors; **C**. Calcic cambisol section at study area, showing the slightly horizon differentiation; **D**. Presence of coal mining wastes located near CM-S3; E; F. Areas with unburned and burned coal mining wastes are present at the site shown in image D

A total of 50 surface soil samples were collected with a maximum depth of 30 cm, using a stainless-steel shovel and after removal of vegetation and coarser materials. The samples were collected at varying distances from the industrial complex, ranging from very close, within 100 m, to distances of approximately 1300 m away from the industrial facilities (Fig. 1D). The sampling sites were defined considering the positioning of the industrial facilities and quarries, and the geological background pursuing the spatial representation. Additionally, it was also respected the access limitations due to local relief conditions and built-up areas (Fig. 2A).

The study area is in a coastal region near a mountain range called *Serra da Boa Viagem* and the elevation range among the collected samples is 181 m, with sample CM-S30 collected at the lowest elevation (14 m) and sample CM-S47 at the highest elevation (195 m). The soil samples predominantly have a sandy texture, as addressed by Michels et al. (2025), and are heterogeneous in colour, varying among yellow/brown, light gray and dark gray groups (Fig. 2B). The samples collected at higher elevations toward the mountain range, farther from the industrial complex, were taken from forested areas. As a result, many of these samples exhibit darker colors, which can be attributed to a higher biomass content and, consequently, a thicker O horizon at these sites.

Some specific field aspects were observed during sampling, including calcic cambisol sections (Fig. 2C), which is the the predominant soil type in the study area, with two characteristic soil horizons. Other aspect is the disposal of coal mining wastes in the place where soil sample CM-S3 was collected, occupaying an area of approximately 15 m^2^ and with less that 1 m high (Fig. 2D). In these coal mining wastes was possible to distinguish a dark grey coloured coaly material (Fig. 2E, F) and also a layer of red coloured material covering the darker material (Fig. 2F). This reddish material is slag with an undefined origin. Two plausible hypotheses are that it could be waste from coal used as fuel in the lime kiln furnaces or residue found in the coal mine resulting from uncontrolled coal combustion during accidental fires. Two samples of unburned coal mining wastes (CM-E1 and CM-E3) and two samples of the reddish burned coal mining wastes (CM-EQ2 and CM-EQ4) were collected for analysis. The analysis of these coal mining waste samples allow the identification and understanding of these materials as a source of contaminants.

In the laboratory, soil samples were homogenized, dried below 40 °C and sieved at 2 mm to remove gravel and organic residues. The coal mining wastes were homogenized and dried below 40 °C. All samples were than quartered to obtain representative portions and grounded to be analyzed for determination of chemical composition. These steps took place in the Santander Laboratory of Geotechnics and Sample Preparation at the University of Coimbra.

### Determination of soil chemical composition

The chemical composition of samples was determined by inductively coupled plasma emission spectrometry/mass spectrometry (ICP-ES/MS) at Bureau Veritas Mineral Laboratories (Canada). The sample preparation and analytical procedures included sorting, labeling, and boxing of samples received as pulps, followed by a four-acid digestion with Ultratrace ICP-MS analysis and heat treatment of soil samples. The data quality was insured using analytical results of certified reference materials (STD OREAS25A-4A and STD OREAS45H and STD OREAS501D), blanks, and random duplicate samples. The results were within the 95% confidence limits of the recommended values given for the certified materials. Bureau Veritas in Vancouver is an accredited laboratory (Accredited Laboratory No. 720). The total Hg content in the samples was determined by the atomic absorption spectrometer (calibrated with a Hg standard) with Zeeman correction for background absorption, using a Lumex RS-915 LAB mercury analyzer device at the University of Coimbra. The samples are stored in the lithotheque of the Department of Earth Sciences at the University of Coimbra under ‘CM-S’ and ‘CM-E’ codes.

### Contamination, statistical analysis and spatial distribution

The Geoaccumulation Index (I_geo_) (Müller, 1969) has been widely employed to assess soil contamination by PTE in numerous studies (Barbieri, 2016; Jaworska & Klimek, 2023; Mazurek et al., 2017). This index is based on the content of each PTE referenced to a specified geochemical background (Kowalska et al., 2018; Müller, 1969). The interpretation of the results enables the determination of the contamination level, categorized into seven distinct classes, as follows: I_geo_ ≤ 0 practically unpolluted (class 0); 0 < I_geo_ < 1 unpolluted to moderately polluted (class 1); 1 < I_geo_ < 2 moderately polluted (class 2); 2 < I_geo_ < 3 moderately to highly polluted (class 3); 3 < I_geo_ < 4 highly polluted (class 4); 4 < I_geo_ < 5 highly to extremely high polluted (class 5); and I_geo_ ≥ 5 extremely high polluted (class 6). The value of the I_geo_ is described by the following equation:

$${I}_{geo}={log}_{2}\left[\frac{Cn}{1.5GB}\right]$$

where *Cn*–concentration of individual heavy metal, *GB* – value of geochemical background and 1.5–constant, to account for natural fluctuations of a given element in the environment, as well as minor anthropogenic influences.

Descriptive statistical analysis, correlation analysis, and principal component analysis (PCA) were conducted using PAST Statistica® and OriginPro® softwares to assess relationships among the variables investigated in the samples and to identify the most prevalent contamination sources. The PCA calculations and corresponding graphical results were obtained using the correlation matrix (covariance matrix of the standardized scaled variables). The number of significant principal components was determined based on the Kaiser criterion (Eigenvalues > 1).

Spatial distribution maps for various measured parameters were generated using the geographical information system software QGIS®. In this instance, inverse distance weighted (IDW) was employed as the interpolation method to generate the maps.

## Results

### Concentration and distribution of trace elements

Table 1 provides the concentration ranges and descriptive statistics for measured trace elements in soil samples, with the complete dataset accessible in the Supplementary Material B. Comparative analysis involves consideration of national reference values for contaminated soils (APA, 2019), Canadian soil guideline (Canadian Environmental Quality Guidelines, 2007) which are worldwide recognized, and composition of soils from Mondego River hydrographic basin (Almeida, 2005), in which Cabo Mondego area is included. These reference values for comparison are also presented in Table 1. Data about concentration of major and minor elements, pH, electrical conductivity (EC) and total organic carbon (TOC) of the studied soil samples, published by Michels et al. (2025), were considered for the integrative characterization of soils.

**Table 1:** Descriptive statistics of soil chemical composition considering trace elements (in mg/kg) and comparison with national and international reference values

Cabo Mondego Soils (n = 50)								National guideline^a^		Canadian Guideline^b^	Soils from Mondego hydrographic basin^c^
	Min	Max	Mean	SD	Median	25 percentile	75 percentile	Agricultural Use	Urban/ Industrial/ Commercial Use		Mean values
Ag	0.01	0.15	0.06	0.03	0.06	0.04	0.08	0.50	0.50		
As	2.80	30.4	11.9	4.53	11.2	8.18	14.3	11.0	18.0	12.0	20.0
Ba	54.0	386	191	67.8	192	157	221	210	220	750	43.0
Be	1.00	4.00	2.08	0.80	2.00	2.00	2.25	2.50	2.50		
Bi	0.13	0.51	0.28	0.08	0.28	0.24	0.32				
Cd	0.01	0.37	0.16	0.10	0.15	0.09	0.23	1.00	1.20	1.40	
Co	1.40	23.1	7.41	4.05	7.25	4.85	8.78	19.0	21.0		7.00
Cr	10.0	91.0	33.0	15.0	31.5	22.7	38.5	67.0	70.0	64.0	17.0
Cs	4.10	17.6	9.66	3.35	9.35	6.68	11.3				
Cu	3.40	32.7	13.4	6.91	12.5	7.95	16.6	62.0	92.0	63.0	14.0
Ga	5.13	22.1	10.2	3.28	10.2	7.50	11.8				
Hf	0.64	2.29	1.36	0.36	1.37	1.10	1.56				
Hg	0.02	0.15	0.06	0.04	0.04	0.03	0.08	0.16	0.27		0.065
In	0.02	0.07	0.04	0.01	0.04	0.03	0.05				
Li	17.5	180	49.4	29.4	43.7	30.8	52.2				
Mn	26.0	2002	454	325	410	263	510				249
Mo	0.15	3.40	0.77	0.56	0.65	0.38	0.92	2.00	2.00		
Nb	3.04	11.2	6.15	1.77	5.96	4.79	7.07				
Ni	3.80	53.0	21.9	10.7	22.1	13.1	27.9	37.0	82.0	50.0	13.0
Pb	10.5	84.7	28.7	12.0	25.7	23.7	30.5	45.0	120	70.0	23.0
Rb	38.7	157	100	30.0	103	79.8	116				
Sb	0.31	3.16	0.73	0.43	0.64	0.53	0.85	1.00	1.30		
Sc	1.50	14.2	5.92	2.51	5.90	4.25	6.58				
Se	0.15	1.90	0.87	0.40	0.80	0.60	1.10	1.20	1.50		
Sn	1.20	9.60	3.11	1.28	3.00	2.40	3.45				
Sr	34.0	342	127.2	81.4	109	63.2	167				19.0
Ta	0.20	1.40	0.62	0.22	0.60	0.48	0.73				
Te	0.03	0.13	0.05	0.03	0.04	0.03	0.08				
Th	3.00	24.2	8.55	3.45	8.05	6.93	9.73				13.0
Tl	0.41	2.40	1.11	0.41	1.03	0.79	1.35	1.00	1.00		
U	1.30	5.40	2.08	0.84	1.80	1.60	2.20	1.90	2.50	23.0	
V	12.0	119	45.5	19.5	44.0	32.7	56.0	86.0	86.0	130	25.0
W	0.60	2.30	1.24	0.36	1.20	0.90	1.50				2.40
Zn	20.1	167	62.8	29.4	55.1	43.1	80.1	290	290	200	53.0
Zr	22.8	80.9	46.8	12.4	46.6	37.5	53.8				
ΣLREE	42.3	255	105	37.8	99.2	78.1	124				
ΣHREE	8.60	60.8	22.7	9.10	22.5	15.9	25.0				
ΣREE	56.1	287	128	44.4	122	94.8	149				

SD = standard deviation; REE = sum of rare earth elements; HREE = sum of heavy rare earth elements; LREE = sum of light rare earth elements; ^a^APA (2019); ^b^Canadian Environmental Quality Guidelines (2007); ^c^Almeida (2005)

The mean concentrations of trace elements follow a descending order as Mn > Ba > Sr > Rb > Zn > Li > Zr > V > Cr > Pb (with minimum values ≥ 10 mg/kg), > Ni > Cu > As > Ga > Cs > Th > Co > Nb > Sc > Sn > U > Be (with minimum values ≥ 1 mg/kg), > Hf > W > Tl > Se > Mo > Sb > Ta > Bi > Cd > Ag > Hg > Te > In (with minimum values < 1mg/kg). The distribution of the dataset was assessed using Shapiro–Wilk's methods (p < 0.05), confirming that most elements do not follow a normal distribution, except for Ag, Ni, Cd, Bi, W, Zr, Hf, Rb, Nb, Cs, and Tl.

**Fig. 3:**
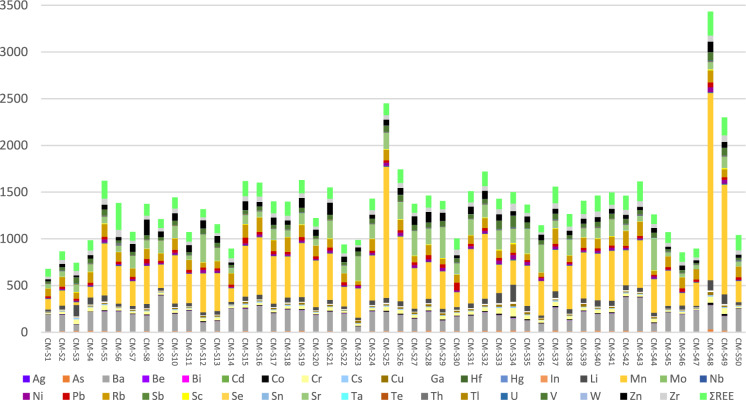
Bar graph illustrating the concentration of trace elements in the soil samples (in mg/kg)

Figure 3 presents the concentration of trace elements in each soil sample, illustrating not only their chemical composition, but also the most evident similarities and differences between the samples, and the sum of selected trace elements. The sum of trace elements varies between 679 mg/kg (CM-S1) and 3433 mg/kg (CM-S48), with a mean value of 1377 mg/kg. Three samples stand out because surpass 2000 mg/kg of the sum of the considered trace elements. These samples are CM-S25, situated close to the industrial complex, and CM-S48 and CM-S49, which are two of the farthest samples from the industrial complex. A common characteristic in these three samples that most stands out compared to the others is the abundance of Mn.

The chemical composition of samples was analyzed grouping them in accordance to lithological background where they were collected (Table 2). Particularly noteworthy is that the maximum and mean values of samples from the area with the Carbonaceous complex and limestones as the geological background present higher values of the majority of PTE (As, Co, Cr, Cu, Mn, Mo, Ni, Se, and V). Regarding major elements, minor elements and EC, the same pattern of higher mean values is observed in the samples associated with the Carbonaceous complex and limestone, except for Na, K, and Ti. The mean of pH in all groups varies little, remaining within the alkaline range. The expected differences in the parameters of soil samples collected in the sandstone background (the only lithology of siliciclastic origin in the study area) are not observed. However, it is noteworthy that these samples have lower mean values for many PTE, but the differences are minimal when compared to other lithologies of carbonate origin.

**Table 2:**
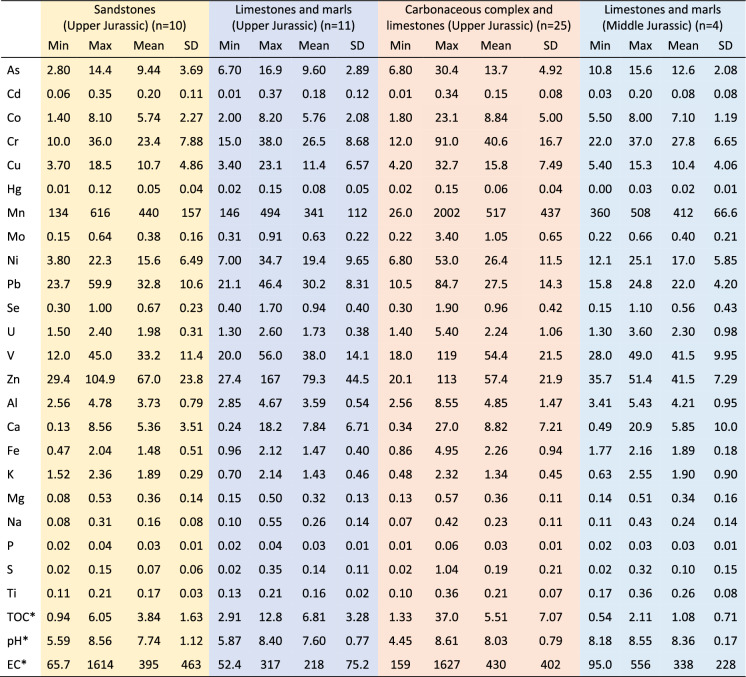
Statistical parameters of soil samples grouped in accordance with the geological background where they were collected

^*^Michels et al. (2025); colors in accordance with the geological map in Fig. 1D

**Fig. 4:**
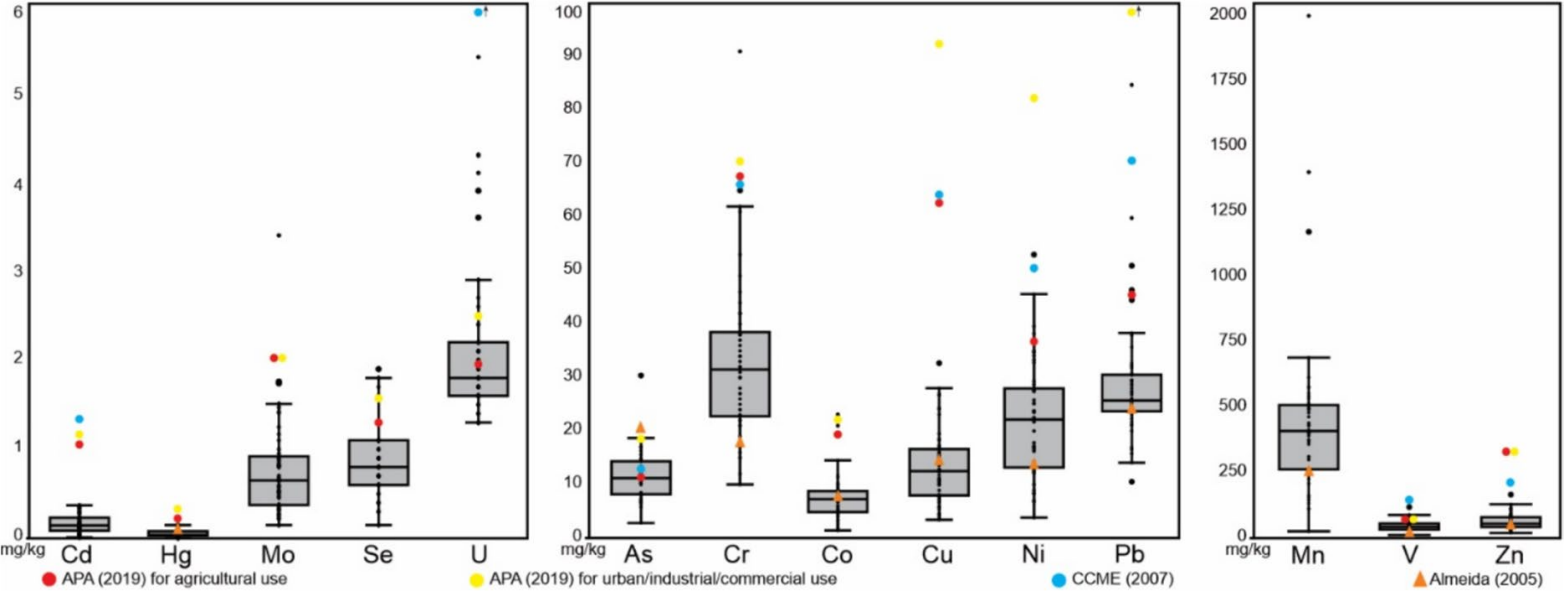
Range of PTE concentration in the soil samples (n = 50) compared with reference values

Regarding the concentrations of PTE in soils, the values were compared with national guideline of contaminated soils for agricultural use and for urban/industrial/commercial use in environmentally sensitive areas (APA, 2019), as well as with international guideline for agricultural use (Canadian Environmental Quality Guidelines, 2007). Additionally, comparisons were also made with mean values from soils of Mondego River hydrographic basin (Almeida, 2005). Figure 4 shows the range of PTE in the investigated soils as well as the described reference values.

In comparison to the Portuguese soils guideline for agricultural use in environmentally sensitive areas, which represents the most conservative reference, 58% of the samples exhibited higher As content dispersed across all regions of the sampling area, 40% displayed higher U contents, 18% had higher Se contents, and 8% presented higher Pb and Ni contents. Additionally, 4% showed higher Co and V contents, and 2% had higher Cr and Mo contents. The values for other PTE fall below the reference value (Cd, Cu, Hg, Zn); there is no reference value for Mn. It is noteworthy that 36 samples exhibit at least one PTE above the reference values for this type of soil use. The elements As, U, Se, and Pb have higher concentrations in sample from the different geological background, while Ni, Co, V, Cr, and Mo have higher concentration in samples with the Carboniferous complex as geological background.

When compared with the national guideline for urban/industrial/commercial use in environmentally sensitive areas, which is less restrictive (except for V that have the same reference value), soils present higher U content in 16% of samples, higher As and Se contents in 10% of samples, higher Co, Cr and Mo contents in 2% of samples. The others PTE values are below the guideline (Cd, Cu, Hg, Ni, Pb, Zn). Twelve samples exhibit at least one PTE above the reference values for this use.

In comparison with the Canadian soils guideline for agricultural use, which is less restrictive than Portuguese guideline, 40% of the samples exhibit higher As values, 4% show higher Cr concentration, and 2% present higher Pb and Ni concentration. The values for other PTE either fall below the reference value (Cd, Co, Cu, Se, U, V, Zn) or lack a reference (Co, Mn, Hg).

The mean concentrations of PTE are also compared with mean values from soils collected in the Mondego River hydrographic basin (Almeida, 2005). The mean values for As, Co, Cu, Hg, Ni, and Pb show similar values. In contrast, the mean values for Cr, Mn, V, and Zn are elevated compared to the values from the Mondego River basin, with Cr, Mn, and V having nearly twice the values of the Mondego River basin. The remaining PTE (Cd, Mo, Se, and U) do not have corresponding mean values for the Mondego River hydrographic basin.

**Fig. 5:**
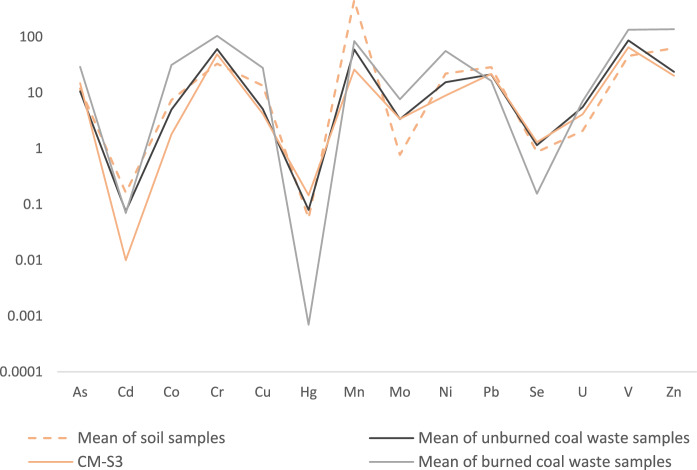
Concentration of PTE (in mg/kg) in the studied soils (mean), soil sample CM-S3, and coal mining wastes samples

Considering the influence that the coal mining waste can cause as a source of contaminantion, principally in sample CM-S3 and surrounding area, the mean concentrations of PTE were evaluated against the soil sample CM-S3, the unburned and burned coal mining waste samples (Fig. 5). Despite the general similarity in the component curves shown in the Fig. 5, it stands out the higher concentration of PTE in burned coal mining waste (except Cd, Hg, Pb, and Se), which is attributed to the combustion process that concentrates less volatile inorganic elements. The lower concentration of Hg and Se is attributed to the volalitilization of these elements during combustion process. It is also observed that the mean of soil samples present the higher concentration of Cd, Mn, and Pb; these samples also have higher concentration of As, Co, Cu, Ni, and Zn when compared to soil sample CM-S3 and unburned coal mining waste samples.

Besides, the composition of soil sample CM-S3 is more similar to the composition of the unburned coal waste, evidencing the influence of these materials in the chemical composition of the soil in the area adjacent to coal mining wastes. The concentration of As, Cr, Hg, Mo, Se, U, and V is higher in sample CM-S3 than the mean of soil samples. From these, As, Mo, Se, and U surpasse the more restrictive reference value for contaminated soil.

### Contamination severity

Table 3 summarizes the I_geo_ results by classes. The detailed calculations are presented in Supplementary Material B. The background values considered correspond to the soil composition from the Mondego River hydrographic basin, which represents the most reliable data on surface soils available at the regional scale (Almeida, 2005), and to the national reference guideline for urban/industrial/commercial use in environmentally sensitive areas (APA, 2019) (Table 1).

**Table 3:**
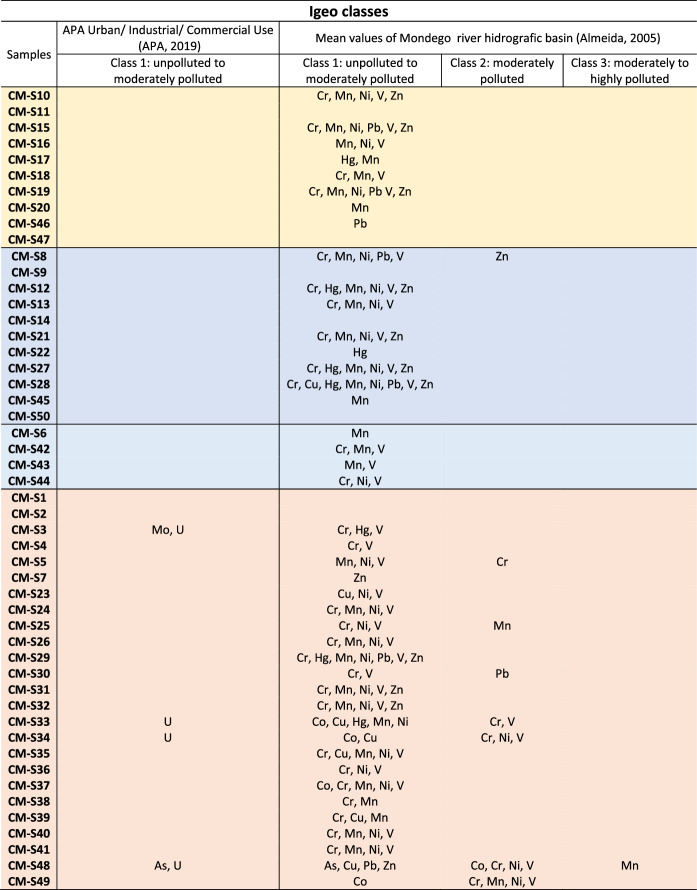
Summary of Igeo results, based on the classes interpretation

Colors in accordance with the geological map in Fig. 1D

It is worth noting that the data from Almeida (2005) does not include available values for Cd, Mo, Se, and U, while the APA dataset does not include available value for Mn. Table 3 exhibits the elements and respective samples that are classified as unpolluted to moderately polluted (class 1), moderately polluted (class 2), and moderately to highly polluted (class 3). Eight samples exhibit moderate pollution levels for PTE (Co, Cr, Mn, Ni, Pb, V, and Zn) based on the regional background. The highest pollution level recorded is class 3, observed exclusively for Mn in sample CM-S48. Based on the national background (APA, 2019), only four samples are not classified as class 0. These samples fall into class 1 for As, Mo, and U.

### Elements association

As most of the data do not exhibit a normal distribution, Spearman correlation analysis was conducted for the PTE values, along with selected major and minor elements and soil physicochemical parameters (pH, EC, and TOC), published by Michels et al. (2025). Data from sample CM-S3 were excluded to reduce bias in the results, as this sample was collected very close to coal mining waste (Fig. 2D) that influences the soil composition.

Table 4 summarizes the correlation coefficients between selected elements and parameters, revealing the identification of two distinct groups of PTE exhibiting significant correlations (> 0.3, *p* < 0.05) and suggesting a potential predominant common source for each group. Group I includes As, Co, Cr, Ni, and V, while Group II comprises Cd, Hg, Se, and Zn. Regarding the correlation of PTE with physicochemical parameters and major and minor elements, notable associations are observed. Elements from Group I show stronger correlations with Al and Fe. On the other hand, Group II exhibits significant correlations with other major and minor elements such as Mg and S. The elements Pb and U stand out because they display weak correlations with most of the other elements, suggesting a potential source distinct from the other elements. Na and K were negatively correlated with most PTE and major elements.

**Table 4:** Spearman correlation coefficients between PTE, major and minor elements and physicochemical properties

	As	Cd	Cr	Co	Cu	Hg	Mn	Mo	Ni	Pb	Se	U	V	Zn	Al	Ca	Fe	K	Mg	Na	S	TOC	pH	EC
As	1																							
Cd	0.19	1																						
Cr	**0.77**	**0.37**	1																					
Co	**0.69**	**0.42**	**0.86**	1																				
Cu	**0.49**	**0.63**	**0.67**	**0.65**	1																			
Hg	0.27	**0.73**	**0.48**	**0.36**	**0.55**	1																		
Mn	**0.40**	**0.44**	**0.48**	**0.67**	**0.38**	0.14	1																	
Mo	**0.51**	**0.38**	**0.79**	**0.61**	**0.66**	**0.56**	0.24	1																
Ni	**0.55**	**0.52**	**0.86**	**0.91**	**0.77**	**0.53**	**0.55**	**0.81**	1															
Pb	0.27	**0.43**	0.23	0.19	0.23	**0.40**	**0.29**	0.09	0.15	1														
Se	0.20	**0.57**	**0.48**	**0.38**	**0.69**	**0.75**	0.02	**0.72**	**0.62**	0.15	1													
U	**0.50**	**− **0.09	0.25	0.24	0.21	**− **0.03	0.13	0.12	0.11	**0.38**	0.03	1												
V	**0.69**	**0.37**	**0.91**	**0.89**	**0.69**	**0.44**	**0.45**	**0.79**	**0.92**	0.16	**0.54**	0.27	1											
Zn	0.26	**0.89**	**0.44**	**0.47**	**0.59**	**0.76**	**0.48**	**0.44**	**0.56**	**0.54**	**0.58**	**− **0.10	**0.41**	1										
Al	**0.79**	0.03	**0.77**	**0.71**	**0.33**	0.04	**0.50**	**0.44**	**0.55**	0.27	0.02	**0.43**	**0.70**	0.14	1									
Ca	0.07	**0.61**	**0.41**	**0.48**	**0.62**	**0.50**	0.28	**0.53**	**0.64**	**− **0.15	**0.62**	**− **0.26	**0.52**	**0.48**	**− **0.01	1								
Fe	**0.85**	0.17	**0.88**	**0.83**	**0.46**	0.23	**0.58**	**0.62**	**0.72**	0.22	0.25	**0.38**	**0.81**	**0.31**	**0.91**	0.18	1							
K	0.00	**− 0.44**	**− 0.34**	**− 0.28**	**− 0.56**	**− 0.54**	0.08	**− 0.53**	**− 0.51**	0.16	**− 0.69**	**0.34**	**− 0.37**	**− 0.36**	0.19	**− 0.73**	**− **0.01	1						
Mg	0.24	**0.68**	**0.49**	**0.61**	**0.67**	**0.50**	**0.45**	**0.46**	**0.69**	0.05	**0.53**	0.02	**0.61**	**0.53**	0.19	**0.81**	**0.32**	**− 0.44**	1					
Na	0.10	**− 0.40**	**− **0.12	**− **0.28	**− 0.42**	**− **0.19	**− **0.18	**− **0.14	**− 0.38**	0.00	**− 0.38**	0.01	**− **0.28	**− **0.29	0.14	**− 0.57**	0.00	**0.53**	**− 0.59**	1				
S	0.12	**0.58**	**0.42**	**0.29**	**0.62**	**0.70**	0.02	**0.65**	**0.52**	**− **0.06	**0.77**	**− **0.17	**0.46**	**0.51**	**− **0.05	**0.75**	0.15	**− 0.69**	**0.62**	**− 0.36**	1			
TOC	**− **0.11	**0.41**	0.09	**− **0.08	0.25	**0.68**	**− **0.29	**0.33**	0.14	0.23	**0.60**	**− **0.12	0.03	**0.49**	**− **0.25	0.14	**− **0.14	**− 0.45**	0.06	**− **0.14	**0.49**	1		
pH	**0.47**	0.11	**0.32**	**0.45**	**0.39**	**− **0.04	**0.44**	0.07	**0.33**	**− **0.03	0.04	**0.32**	**0.34**	0.07	**0.34**	0.28	**0.39**	0.00	**0.36**	**− **0.16	**− **0.03	**− 0.45**	1	
EC	**0.32**	**0.38**	**0.38**	**0.40**	**0.55**	0.25	0.19	**0.37**	**0.44**	0.00	**0.34**	0.07	**0.43**	0.23	**0.31**	**0.51**	**0.32**	**− **0.23	**0.67**	**− **0.26	**0.50**	**− **0.06	0.13	1

Bold values are significant at *p* < 0.05

Regarding the association of physicochemical parameters with the two defined groups of elements, it is notable that pH exhibits significant positive correlations with elements of Group I (> 0.32, p < 0.05). In contrast, elements from Group II show weak correlations with pH (< 0.11, *p* < 0.05). When comparing the elements of Group II with TOC, it displays significant positive correlations (> 0.41, *p* < 0.05), whereas Group I shows weak correlations (< 0.14, *p* < 0.05). These results indicate that pH is preferentially correlative with Group I, while TOC is preferentially associated with Group II. EC shows similar correlations with elements from both groups. The pH demonstrates a significant negative correlation with TOC (− 0.45, *p* < 0.05), as is commonly observed in soils under natural conditions due to their inherent dynamics (Xu & Zhang, 2021).

**Fig. 6:**
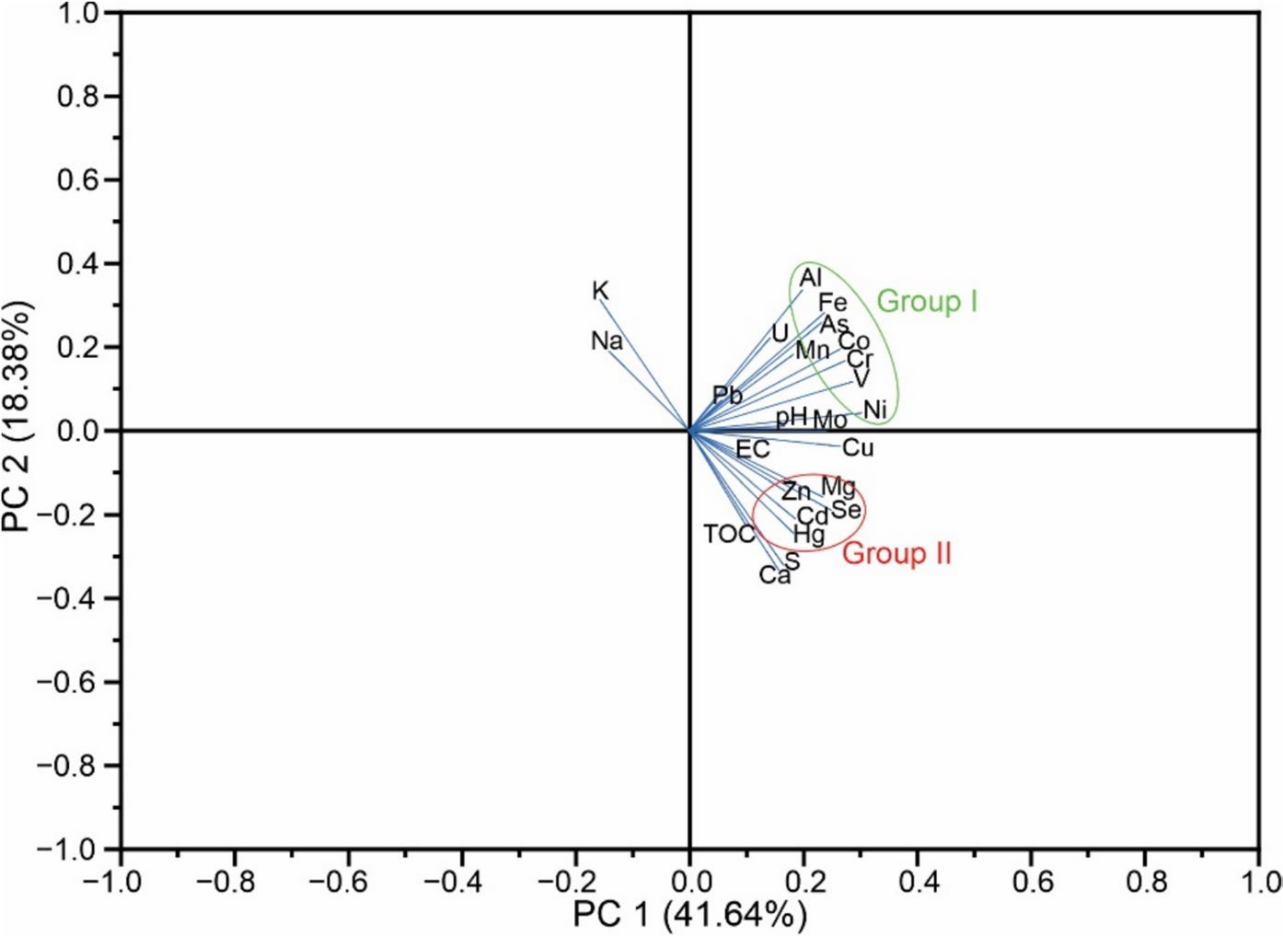
Biplot for the best two factors: principal component 1 (PC1) versus principal component 2 (PC2)

The PCA analysis extracted five principal components, explaining 80.85% of the overall variance in the studied materials. The statistical analysis of data using PCA based on the representation of the first two components (which captured 60.01% of the total variability) is consistent with the observed in the correlation analysis, as illustrated in Fig. 6. An association is observed in the upper right quadrant, represented by As, Co, Cr, Mn, Ni, and V (Group I). Another significant association is observed in the lower right quadrant consisting of Cd, Hg, Se, and Zn (Group II).

Upon comparison with major elements and physicochemical parameters, Group I shows associations with Al, Fe, and pH, while Group II is more closely associated with Ca, Mg, S and TOC. Additionally, a noticeable correlation between Na and K is observed on the left side, not revealing an association with both Groups I and II.

### Spatial distribution of PTE

**Fig. 7:**
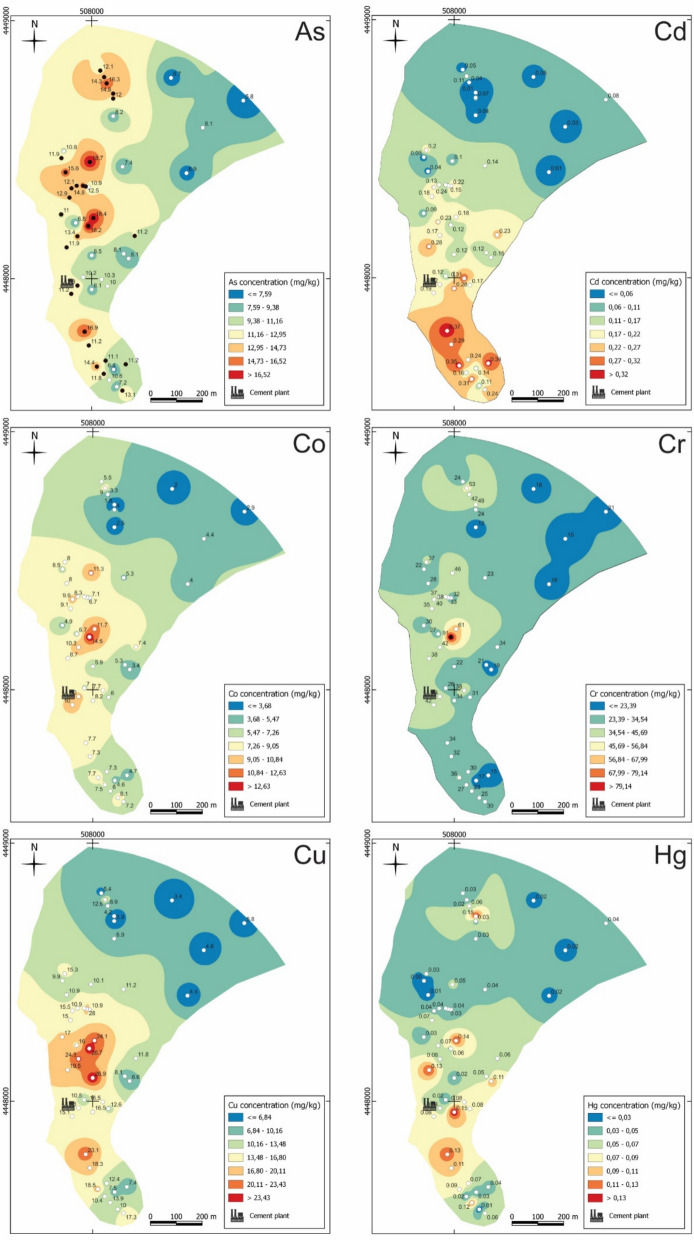
Spatial distribution of As, Cd, Co, Cr, Cu and Hg concentration in the soils of the study area. The white points indicate values below and the black points indicate values above the national guideline for contaminated soils

**Fig. 8:**
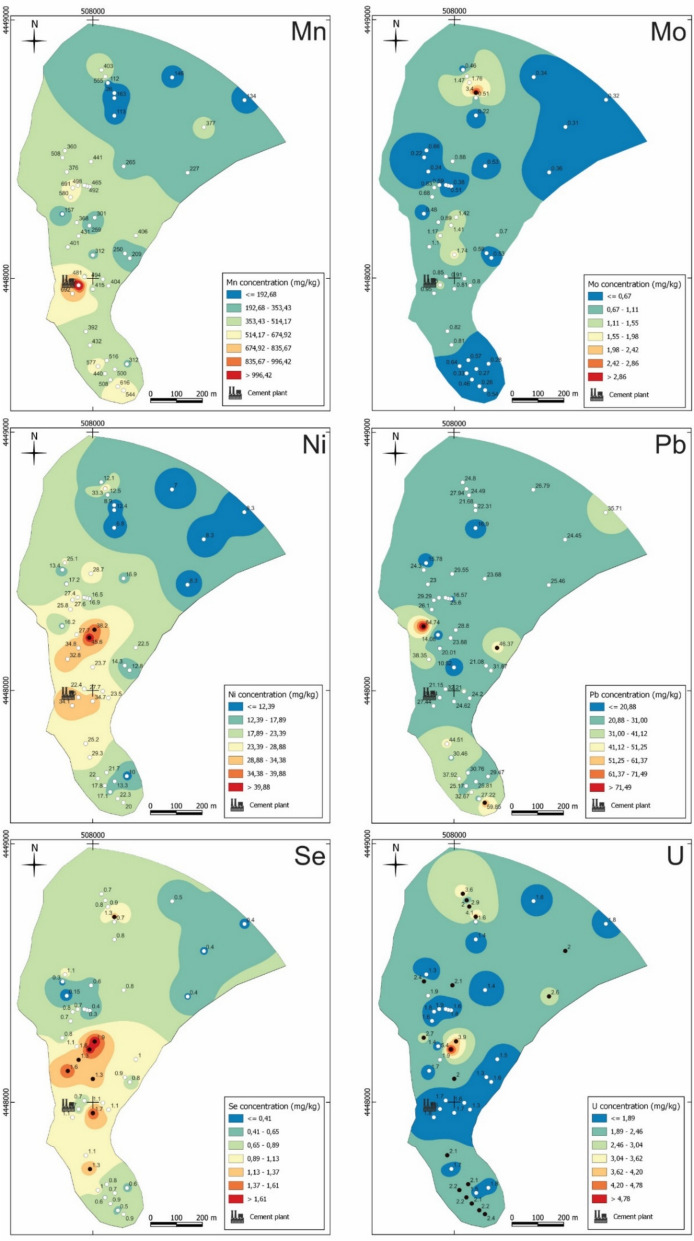
Spatial distribution of Mn, Mo, Ni, Pb, Se and U concentration in the soils of the study area. The white points indicate values below and the black points indicate values above the national guideline for contaminated soils

**Fig. 9:**
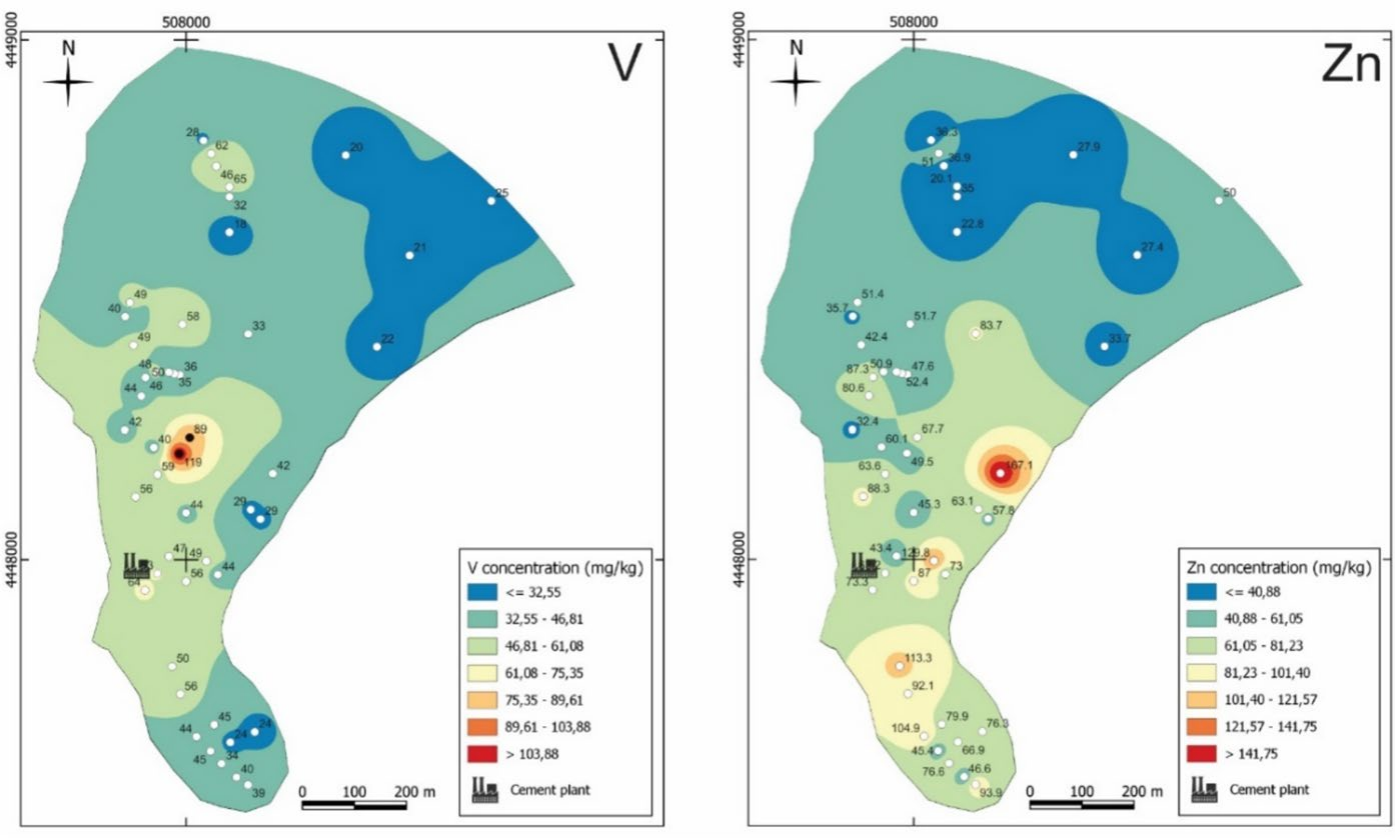
Spatial distribution of V and Zn concentration in the soils of the study area. The white points indicate values below and the black points indicate values above the national guideline for contaminated soils

Figures 7, 8 and 9 show the spatial interpolation mapping of PTE distributions using the IDW method. The white sampling points on the maps indicate values below and the black points indicate values above the national guideline for soils designated for agricultural use (APA, 2019). The maps were developed considering samples located within a 1000 m radius of the industrial facilities, therefore excluding samples CM-47, CM-S48, and CM-S49. These three samples were collected in higher areas at the greatest distance from the industrial complex (between 1000 and 1500 m away), currently designated for forestry. They exhibit distinct macroscopic parameters, and were excluded from this analysis to prevent distortions in the interpolations.

The most discernible pattern in the maps show higher concentrations of As, Co, Cr, Ni, Se, and V concentrated around the immediate vicinity of the industrial facilities towards the north, although high As levels are also observed to the south. Another pattern reveals higher concentrations of Cd, Cu, Hg, and Zn distributed around the immediate vicinity of the industrial facilities towards the south near the facilities dedicated to cement and lime processing in the past.

The samples located at the northern/northeastern and southern extremities of the study area commonly have lower concentration of PTE, except sample CM-S3 (collected near the coal mining wastes), for which concentration of As, Mo, and Se stands out.

The Mn map exhibits an anomalous high value in CM-S25, situated near the industrial access road area in close proximity to the facilities, reaching 3.5 times the mean value for Mn. Similarly, this sample displays an elevated EC value surpassing the national reference value for contaminated soils (APA, 2019), and indicating a potential link between the high Mn concentration and the elevated EC value.

The Pb map displays significantly elevated values which are above the Portuguese reference value for contaminated soils (APA, 2019) in distinct regions (CM-S8, CM-S15, and CM-S30). The spatial distribution of U exhibits a different pattern compared to the others PTE, with very similar values dispersed throughout all sectors of the study area (Fig. 8). The samples near the industry show lower values.

## Discussion

The results confirm the hypothesis of soil contamination by PTE in the vicinity of the industrial facilities. Atmospheric emissions from fuel combustion within the industrial complex played a critical role in dispersing contaminants across soils and possibly into the ocean, driven by wind atmospheric transport. Additional sources of contamination are linked to fuel and coal mining waste disposal in specific sites.

Regarding the association of elements, the components of Group I are linked with the mineral fraction of soils. The elements such as As, Co, Cr, and V may be predominantly associated with geogenic sources in the study area. This association is likely due to their strong affinity with Fe and Al present in common soil oxides, which have a high potential for absorption by the clay fraction (Kabata-Pendias, 2011). On the other hand, it is noteworthy that Cd, Hg, Se, and Zn (Group II) are more closely associated with the organic matter present in the soils, as evidenced by the correlation and PCA analysis. The enrichment of the elements of Group II in the soil samples around the industrial site suggest a probable association with anthropogenic sources related to emissions from the industry. Additionally, assuming that the burned coal waste samples are originated from coal used as fuel in facilities, the lower concentrations of Cd, Hg, and Se in these samples could be linked to the enriched levels of these elements in the soils surrounding the industrial complex. It may be attributed to the high volatility of these elements, along with carbon, during the coal combustion process (Ullah et al., 2019).

According to the guideline for soils in Portugal (APA, 2019), 36 samples exhibit PTE levels exceeding the most restrictive reference values. Based on APA (2019) and APA (2022), these samples are classified as contaminated, as they contain at least one PTE with concentration above the established references. The contaminated samples originate from all sectors of study area, and those containing higher concentration of elements above the reference levels have the Carbonaceous complex as their geological background. For soils designated for urban/industrial/commercial use, twelve samples exhibit at least one PTE above the reference values. Almost all of these samples (except CM-S12 and CM-S50) have the Carbonaceous complex as their geological background.

The most critical PTE observed is As, given that its concentrations exceed reference values in 29 samples across the study area, except in samples northeast of the industrial facilities. Arsenic is widely dispersed throughout the environment, particularly related to sulfides such as arsenopyrite and pyrite, which are commonly found in sedimentary formations rich in organic matter, notably shale, coal, and peat deposits (Kabata-Pendias, 2011; Rouhani et al., 2023). Anthropogenic activities that contribute to As contamination in soils include emissions from coal-fired power plants and mining related operations (Aide et al., 2016 and references therein). The elevated occurrence of As in samples located close and predominantly south of the industrial complex, as well as in those close the coal mining waste, suggests the involvement of anthropogenic sources. However, As content may also originate from geogenic sources in the others enriched samples, indicating a combination of both geological and anthropogenic contributions. Correlation coefficients and PCA analysis facilitated the categorization of the elements based on their similarities, offering valuable insights into alternative sources of PTE in the examined soils. Notably, As demonstrates interrelationships with the major elements Fe and Al, and with pH. As previously stated, Fe and Al are prone to accumulation in soils, due to their limited mobility and their association with oxides resulting from natural weathering processes (Kabata-Pendias, 2011; Santos et al., 2020; Sposito et al., 2008). Moreover, this association may indicate the presence of pyrite and other sulfides originating from coal (Finkelman et al., 2018). Elevated concentrations of Al, Fe, as well as As, are notably observed in samples sourced from Carbonaceous complex geological background, the rocks where coal were historically extracted in the Cabo Mondego coal mine.

The second PTE that most exceed the reference values is U, although the values show minimal overall variation, remaining close to the reference of 2 mg/kg (APA, 2019). All sectors of the study area present values above guideline, except samples close to industrial facilities. Uranium in coal deposits can be found in association with organic matter and mineral constituents (Finkelman et al., 2018), however U is not often cited associated with soil contamination from cement/lime industries. Studies from Argentina and South Africa (Bermudez et al., 2010; Olowoyo et al., 2015) cite similar mean concentrations of U, compared to the present study. Therefore, the concentration of U in the samples may be linked to geogenic sources.

Selenium concentrations exceed the reference values established by the APA in seven samples surrounding the industrial facilities, and in sample CM-S3 (Fig. 8). Environmental Se contamination can arise from both natural and anthropogenic mechanisms (Ullah et al., 2022). Among non-natural sources, coal combustion is one of the primary causes of Se contamination, as Se is one of the most volatile trace elements in coal, primarily emitted as vapor in the forms of SeO₂ and SeO₃ (Ullah et al., 2019). The data suggest that the origin of the elevated Se values is the emissions from the industrial complex, as coal was the primary fuel for heating the kilns during a significant period of industrial operation in Cabo Mondego (Campos, 1983).

The four samples that exceed reference values for Pb are distributed within the study area and tend to be located near roads both inside and outside the industrial complex. Lead is not naturally prevalent in soils but can be introduced through anthropogenic activities such as industrial processes, mining, lead-based paint, and leaded fuels (Ismail et al., 2023; Olowoyo et al., 2022). Considering the observed, Pb contamination in these samples can be due to the disposal or leakage of unidentified leaded fuels into the environment. In this context, it is worth to mention that the kilns of the cement factory in Cabo Mondego was fueled with fuel oil from the 1960s until its closure in 1986 (Campos, 1983).

The anomalous Mn value observed in CM-S25 (Fig. 8) is attributed to localized unidentified contamination associated with the industrial complex, as this sample was collected beside an internal road in close proximity to the buildings. The hypothesis finds support in the spatial distribution of EC values (Michels et al., 2025), which exhibits a similar pattern with the Mn abnormal value. Further reinforcement for these observations comes from related research conducted by Ameraoui et al. (2017), which documented significant increases in EC values in areas surrounding cement factories. It is noteworthy that other elevated Mn values tend to appear in samples near the industrial facilities and are most likely attributed to atmospheric emissions. Samples CM-S33 and CM-S34, situated very close to each other near the slope of the oldest quarry in Cabo Mondego (Goulão, 2016) and adjacent to the industrial facilities, exhibit higher concentrations of As, Cr, Ni, Se, and V. The elevated levels of these PTE may be associated with atmospheric emissions from the industry or the deposition of industrial residues.

The I_geo_ results corroborate the previously mentioned findings for samples CM-S8, CM-S25, CM-S30, CM-S33, and CM-S34, which are classified as moderately polluted with respect to Co, Cr, Mn, Ni, Pb, V, and Zn. The Class 2 and Class 3 indices observed in samples CM-S48 and CM-S49 appear to be influenced by the physicochemical properties of the soils in these areas. Notably, these samples were collected at higher elevations and at greater distances from the industrial complex, within a region currently designated for forestry. The thicker O horizon and the consequently high content of organic matter in these forested areas likely act as a filter, effectively binding PTE and restricting their migration into deeper soil layers (Mazurek et al., 2017). Considering that the majority of the study area is dominated by well-drained Cambisols, the collection site for these two samples exhibits markedly distinct characteristics compared to the other locations.

Besides the highlighted soil samples and areas considered contaminated due to high concentrations of PTE exceeding guideline and attributed to anthropogenic factors and geogenic characteristics, sample CM-S3 reavels different geochemical fingerprint when compared with the other soil samples, standing out the concentration of Mo. The contamination of this soil sample caused by coal mining waste is also indicated by the abnormal values of pH, EC, and TOC (Michels et al., 2025). The correlation coefficients between TOC and Mo (0.33, *p* < 0.05), TOC and Hg (0.68, *p* < 0.05) and TOC and Se (0.60, *p* < 0.05) indicate their preferential association with organic matter in coaly materials (Finkelman et al., 2018). This supports that the antropogenic source of Mo in sample CM-S3 is related with the coal waste disposal and soil contamination with Hg and Se may be related with volatilization and dispersion of these PTE during coal combustion in kilns.

## Conclusions

A total of 50 soil samples were collected in the vicinity of a deactivated industrial complex, quarries, and a former coal mine in the protected area of Cabo Mondego (Portugal) to determine the levels and spatial distribution of PTE. The concentration of elements reveals that As exceed the national reference value for soils in environmentally sensitive areas in 58% of the samples, followed by U in 40% of samples, Se in 18% of the samples, Pb and Ni in 8% of the samples, Co and V in 4% of the samples, Cr and Mo in 2% of the samples. Despite the rugged terrain, cliffs, and longstanding potential contaminant activities that make the area highly complex, the I_geo_ values, statistical and spatial distribution analyses revealed geogenic sources of As distribution in soil samples from areas with the Carbonaceous complex geological background. Additionally, the concentrations of U found in soil samples dispersed throughout the study area are also associated with natural sources. Regarding anthropogenic influence, three main sources of soil contamination were identified: atmospheric emissions from fossil fuel combustion in the industrial complex are primarily linked to contamination by As and Se, with a few samples also showing contamination by Cr, Ni, and V; the disposal of coal mining waste are associated with contaminated samples by As, Mo, and Se; and the disposal of fuels at specific locations are associated with contaminated samples by Pb. The values for other PTE (Cd, Cu, Hg, Mn, Zn) either fall below the reference values; however, these elements also show enriched concentrations in soil samples closer to the industrial complex, indicating an influence of industrial atmospheric emissions. This study outlines the geochemical alterations in the soils of Cabo Mondego, contributing to a better understanding of the vulnerabilities associated with this Natural Monument, which is particularly relevant to the ongoing Atlantic Geopark project.

## Supplementary Information

Below is the link to the electronic supplementary material.

Supplementary file1 (DOCX 27 KB)

Supplementary file2 (XLSX 78 KB)

## Data Availability

Data is provided within the manuscript or supplementary information files.
