# Supplementary material for: The problematic of soil contamination by industries in a protected area in Portugal
Source: Environ Geochem Health. 2025 Apr 9;47(5):160. doi: 10.1007/s10653-025-02445-5 (PMC11982105; doi:10.1007/s10653-025-02445-5)
Supplement: Supplementary file 1 — Supplementary file1 (DOCX 27 KB) [file 10653_2025_2445_MOESM1_ESM.docx]

Supplementary material A: Concentration of potentially toxic elements in soils affected by cement plants around the world (in mg/kg).

| Origin | As | Cd | Co | Cr | Cu | Hg | Mn | Ni | Pb | U | V | Zn | References |
| --- | --- | --- | --- | --- | --- | --- | --- | --- | --- | --- | --- | --- | --- |
| Cabo Mondego soils (n = 50) | 2.8 – 30.4 (11.87) | 0.01 – 0.37 (0.16) | 1.4 – 23.1 (7.40) | 10 - 91 (33.02) | 3.4 – 32.7 (13.39) | 0 – 0.151 (0.05) | 26 - 2002 (454.22) | 3.8 - 53 (21.96) | 10.52 – 84.74 (28.70) | 1.3 – 5.4 (2.07) | 12 - 119 (45.52) | 20.1 – 167.1 (62.84) |  |
| Algeria | _ | 1.75 | _ | _ | 43.96 | _ | _ | _ | 145.46 | _ | _ | 282.82 | Ameraoui *et al*. (2017) |
| Argentina | 5.0 – 9.7 (7.6) | _ | _ | 37.7 – 91.5 (50.2) | 0.59 – 6.10 (3.74) | _ | 173 - 399 (281) | 1.43 – 7.17 (3.19) | 6.41 – 9.50 (7.7) | 1.7 – 4.4 (3.1) | _ | 64.6 - 156 (92.5) | Bermudez *et al*. (2010) |
| Brasil | _ | 0.05 – 0.06 | 1.85 – 5.92 | 19.64 – 35.53 | 7.03 – 10.87 | _ | 205.51 – 490.76 | 3.12 – 4.77 | 13.59 – 21.04 | _ | _ | 13.23 – 27.02 | Silva *et al*. (2021) |
| China | _ | 0.8 | 22.3 | 235.8 | 71.08 | _ | 1796.9 | 88.4 | 56.6 | _ | _ | 216.5 | Liu *et al*. (2019) |
| Ghana | _ | _ | 54.54 | 961.24 | 27.97 | _ | 544.92 | 245.26 | 13.13 | _ | _ | 35.02 | Addo *et al*. (2012) |
| India | _ | _ | _ | 16.59 | 35.58 | _ | 128.1 | 18.39 | 11.49 | _ | _ | _ | Kaur *et al*. (2019) |
| Iran | _ | 0.80 – 8.50 (1.96) | _ | _ | _ | _ | _ | 31.50 – 124.15 (88.09) | 23.55 – 41.55 (30.74) | _ | _ | _ | Amiri *et al*. (2021) |
| Iran | _ | _ | _ | 65 - 201 | 11 - 233 | _ | _ | 41 - 97 | 27 - 133 | _ | _ | 67 - 402 | Jafari *et al*. (2019) |
| Iraq | _ | 0.1 | _ | _ | 23.6 | _ | _ | 112.8 | 92.1 | _ | _ | 63.8 | Khwedim *et al*. (2015) |
| Jamaica | _ | 5.24 | _ | 57.21 | _ | _ | _ | _ | 31.47 | _ | _ | 132.03 | Mandal & Voutchkov (2011) |
| Nigeria | _ | _ | _ | 76.4 | 5.03 | _ | 466 | 29.1 | 19.3 | _ | _ | 10.1 | Kolo *et al*. (2018) |
| Saudi Arabia | _ | _ | _ | 88.33 - 200 (138.67) | 12.3 - 64 (33.48) | _ | _ | 18.3 – 68.3 (41.22) | 280.4 – 586.4 (460.15) | _ | _ | 15.2 – 89.9 (54.41) | El-Sherbiny *et al*. (2019) |
| South Africa | 8.35 | 0.2 | _ | 257.85 | 82.56 | _ | 230.45 | 109.5 | 121.55 | 1.8 | _ | 137 | Olowoyo *et al*. (2015) |
| Spain | _ | 0.3 | 4 | 10.3 | 27.6 | _ | 213.7 | 11.3 | 16.4 | _ | _ | 38.2 | Schumacher *et al* (2002) |
| Spain | 12 | 0.29 | 8.73 | 26.8 | 38.1 | 0.09 | 468 | 19 | 48.7 | _ | 38 | 98.1 | Rovira *et al*. (2011) |
| Turkey | _ | 0.89 | _ | 194.8 | 36.7 | _ | _ | 285.1 | 0.7 | _ | _ | 178.8 | Saltali *et al*. (2018) |
| Turkey | _ | 0.86 – 1.71 (1.39) | 0.12 – 5.76 (1.18) | 11.35 – 27.42 | 15.65 – 81.85 (31.20) | _ | 212.40 - 1199 (499.68) | 15.49 – 130.65 (41.17) | 7.51 – 82.66 (22.08) | _ | _ | 31.34 – 156.8 (50.91) | Parlak *et al*. (2023) |

Min - Max (mean) or mean values.

**References**

Addo, M.A., Darko, E.O., Gordon, C., Nyarko, B.J.B., Gbadago, J.K., Nyarko, E., Affum, H.A., Botwe, B.O. (2012). Evaluation of heavy metals contamination of soil and vegetation in the vicinity of a cement factory in the Volta Region, Ghana. International Journal of Science and Technology, 2(1), 40-50.

Ameraoui, S., Boutaleb, A., Souiher, N., Berdous, D. (2017). Investigation of potential accumulation and spatial distribution of heavy metals in topsoil surrounding the cement plant of Meftah (southeastern Algiers region, Algeria). Arabian Journal of Geosciences, 10, 1-12. <https://doi.org/10.1007/s12517-017-3245-0>

Amiri, H., Daneshvar, E., Azadi, S., Azadi, S. (2022). Contamination level and risk assessment of heavy metals in the topsoil around cement factory: A case study. Environmental Engineering Research, 27(5), 1-7. https://doi.org/10.4491/eer.2021.313

Bermudez, G.M., Moreno, M., Invernizzi, R., Plá, R., Pignata, M.L. (2010). Heavy metal pollution in topsoils near a cement plant: The role of organic matter and distance to the source to predict total and HCl-extracted heavy metal concentrations. Chemosphere, 78(4), 375-381. <https://doi.org/10.1016/j.chemosphere.2009.11.012>

El-Sherbiny, M.M., Ismail, A.I., El-Hefnawy, M.E. (2019). A preliminary assessment of potential ecological risk and soil contamination by heavy metals around a cement factory, western Saudi Arabia. Open Chemistry, 17(1), 671-684. https://doi.org/10.1515/chem-2019-0059

Jafari, A., Ghaderpoori, M., Kamarehi, B., Abdipour, H. (2019). Soil pollution evaluation and health risk assessment of heavy metals around Douroud cement factory, Iran. Environmental Earth Sciences, 78, 1-9. https://doi.org/10.1007/s12665-019-8220-5

Kaur, M., Bakshi, M., Bhardwaj, R., Verma, N. (2019). Soil and Air Pollutant Loads on Plants from a Cement factory in Haridwar District, Uttarakhand. Indian Journal of Forestry, 42(3), 263-271.

Kolo, M.T., Khandaker, M.U., Amin, Y.M., Abdullah, W.H.B., Bradley, D.A., Alzimami, K.S. (2018). Assessment of health risk due to the exposure of heavy metals in soil around mega coal-fired cement factory in Nigeria. Results in physics, 11, 755-762. https://doi.org/10.1016/j.rinp.2018.10.003

Khwedim, K., Meza-Figueroa, D., Hussien, L.A., Del Río-Salas, R. (2015). Trace metals in topsoils near the Babylon Cement Factory (Euphrates River) and human health risk assessment. Environmental Earth Sciences, 74, 665-673. https://doi.org/10.1007/s12665-015-4071-x

Liu, Y.H., Wang, X.S., Guo, Y.H., Mao, Y.M., Li, H. (2019). Association of black carbon with heavy metals and magnetic properties in soils adjacent to a cement plant, Xuzhou (China). Journal of Applied Geophysics, 170, 103802. <https://doi.org/10.1016/j.jappgeo.2019.06.018>

Mandal, A., & Voutchkov, M. (2011). Heavy metals in soils around the cement factory in Rockfort, Kingston, Jamaica. International Journal of Geosciences, 2(1), 48. DOI: 10.4236/ijg.2011.21005

Olowoyo, J.O., Mugivhisa, L.L., Busa, N.G. (2015). Trace metals in soil and plants around a cement factory in Pretoria, South Africa. Polish Journal of Environmental Studies, 24(5), 2087-2093. <https://doi.org/10.15244/pjoes/43497>

Parlak, M., Everest, T., & Tunçay, T. (2023). Spatial distribution of heavy metals in soils around cement factory and health risk assessment: a case study of Canakkale-Ezine (NW Turkey). Environmental Geochemistry and Health, 45, 5163-5179. https://doi.org/10.1007/s10653-023-01578-9

Rovira, J., Mari, M., Nadal, M., Schuhmacher, M., Domingo, J.L. (2011). Levels of metals and PCDD/Fs in the vicinity of a cement plant: assessment of human health risks. Journal of Environmental Science and Health, Part A, 46(10), 1075-1084. DOI: 10.1080/10934529.2011.590383

Saltalı, K., Gündoğan, R., Faruk, Ö., Zekeriya, D., Tuğrul, K. (2018). Chimney dusts effects of cement factory on heavy metal contents of Narlı plain’s soils. Journal of Soil Water, 7(1), 11-20. DOI: 10.21657/topraksu.410113

Schuhmacher, M., Bocio, A., Agramunt, M.C., Domingo, J.L., De Kok, H.A.M. (2002). PCDD/F and metal concentrations in soil and herbage samples collected in the vicinity of a cement plant. Chemosphere, 48(2), 209-217. <https://doi.org/10.1016/S0045-6535(02)00042-5>

Silva, T.A.D.C., Paula Jr, M.D., Silva, W.S., Lacorte, G.A. (2021). Deposition of potentially toxic metals in the soil from surrounding cement plants in a karst area of Southeastern Brazil. Conservation, 1(3), 137-150. <https://doi.org/10.3390/conservation1030012>
